# Supplementary material for: Implicit model to capture electrostatic features of membrane environment
Source: PLoS Comput Biol. 2024 Jan 22;20(1):e1011296. doi: 10.1371/journal.pcbi.1011296 (PMC10833867; doi:10.1371/journal.pcbi.1011296)
Supplement: S1 Text — Fig A. WALP35 peptide at the center of the lipid membrane. (a) The total energy by F23 and ΔGw,laa using F19 and F23 calculated as a function of tilt angles. (b) The net energy components due to the presence of the lipid membrane as a function of tilt angle calculated using F19(including ΔGw,l), F23 (including ΔGw,laa,ΔGlipidaa and M12 (including hydrophobicity, solvation, and knowledge terms). WALP39 peptide in the lipid membrane. (c) The per-residue ΔGw,laa calculated using F23 and F19 for WALP39 at a depth = 10Å, tilt-angle = 90° and azimuthal-angle = 0°. (d) The total energy by F23 and ΔGw,laa using F19 and F23 for WALP39 calculated as a function of tilt angles. (e) The net energy components due to the presence of the lipid membrane at depth = 0Å and as a function of tilt angle calculated using F19, F23 and M12. The arrows show the corresponding axes for the plot. The ones for which arrows are not shown have values represented in the other direction. Fig B. Comparing the energy landscape for WALP25 and WALP35 by M12 at the center of the lipid membrane. The M12 total energy and other membrane-based energy terms were calculated for WALP25 and WALP35 peptides as a function of tilt angle at a depth = 0Å and minimized over rotation angle. The different score terms are as follows: (a) The total energy due to the membrane environment includes hydrophobicity, solvation, and knowledge terms. (b) the knowledge term is a statistical measure of the propensity of a residue to be at a particular membrane depth. (c) hydrophobicity energy and (d) solvation energy. Fig C. Energy landscape of influenza A M2 peptide (pdb: 1mp6). (a) Comparing the native tilt angle (gray) with that calculated by F19 (blue) and F23(magenta and light pink). The Asp and c-term Leu for which the ΔGw,laa is modified by F23 are shown in sticks. The total energy landscape of 1mp6 is shown as calculated by (b) F19 and (c) F23 as a function of depth in the membrane and tilt angle. The energy at each tilt an [file pcbi.1011296.s001.pdf]

Supplementary Information Appendix

S1 Text. Implicit model to capture electrostatic features of membrane environment

Rituparna Samanta[1] **,Jeffrey J. Gray**[1,2,3*1]

[1]Department of Chemical and Biomolecular Engineering, The Johns Hopkins University, Baltimore, Maryland, United States of America.; [2]Program in Molecular Biophysics, The Johns Hopkins University, Baltimore, Maryland, United States of America.; [3]Sidney Kimmel Comprehensive Cancer Center, The Johns Hopkins School of Medicine, Baltimore, Maryland, United States of America.

*To whom correspondence should be addressed. E-mail: jgray@jhu.edu

Figures. A to F
Tables A to C
References

1. Supplementary Results

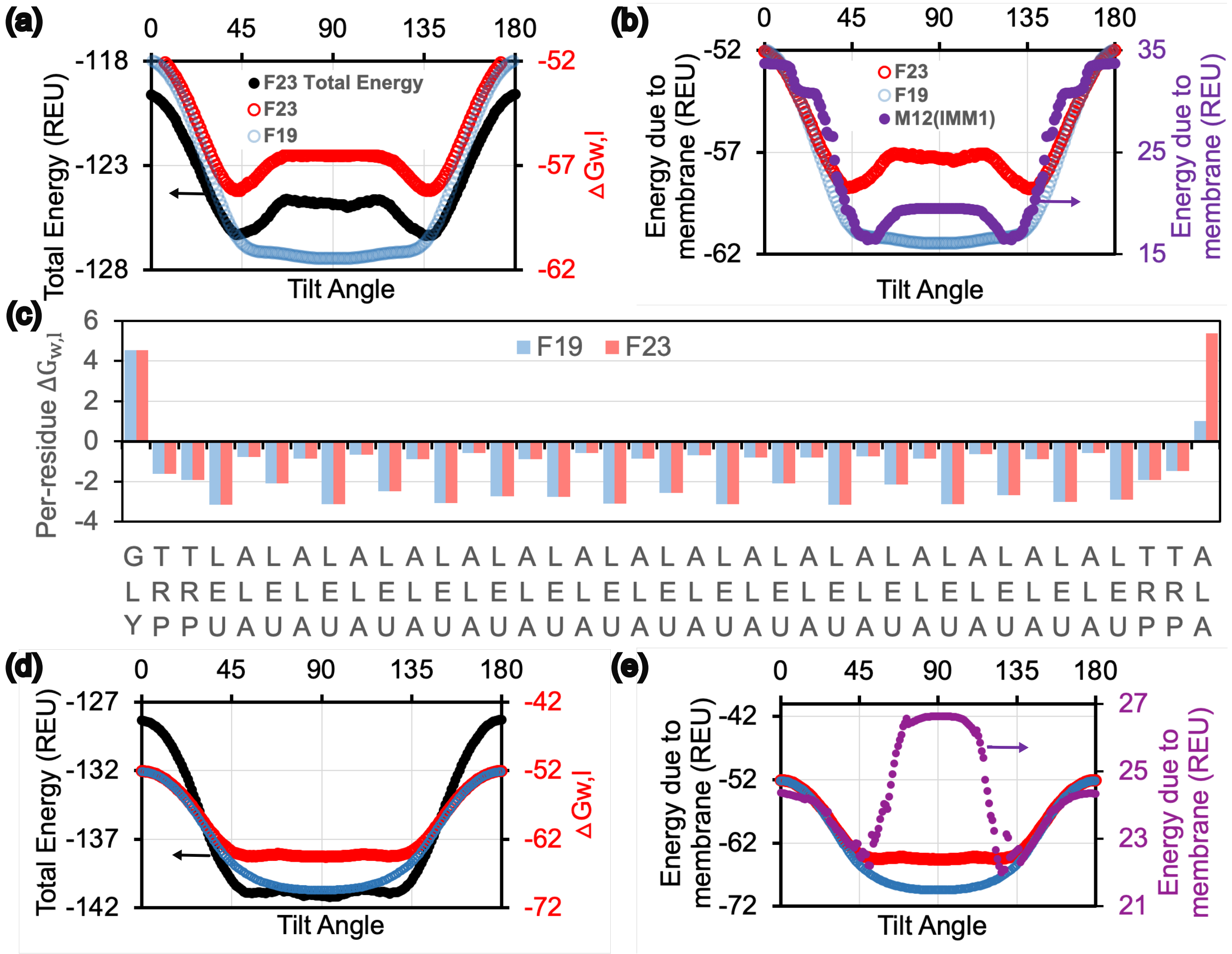

Fig. A. WALP35 peptide at the center of the lipid membrane. (a) The total energy by F23 and $\Delta G^{aa}_{w,l}$ using F19 and F23 calculated as a function of tilt angles. (b) The net energy components due to the presence of the lipid membrane as a function of tilt angle calculated using F19(including $\Delta G_{w,l}$), F23 (including $\Delta G^{\text{aa}}_{w,l}$,$\Delta G^{\text{aa}}_{\text{lipid}}$ and M12 (including hydrophobicity, solvation, and knowledge terms). **WALP39 peptide in the lipid membrane.**(c) The per-residue $\Delta G^{aa}_{w,l}$ calculated using F23 and F19 for WALP39 at a depth = 10Å, tilt-angle=$90°$ and azimuthal-angle = $0°$. (d) The total energy by F23 and $\Delta G^{aa}_{w,l}$ using F19 and F23 for WALP39 calculated as a function of tilt angles. (e) The net energy components due to the presence of the lipid membrane at depth = 0Å and as a function of tilt angle calculated using F19, F23 and M12. The arrows show the corresponding axes for the plot. The ones for which arrows are not shown have values represented in the other direction.

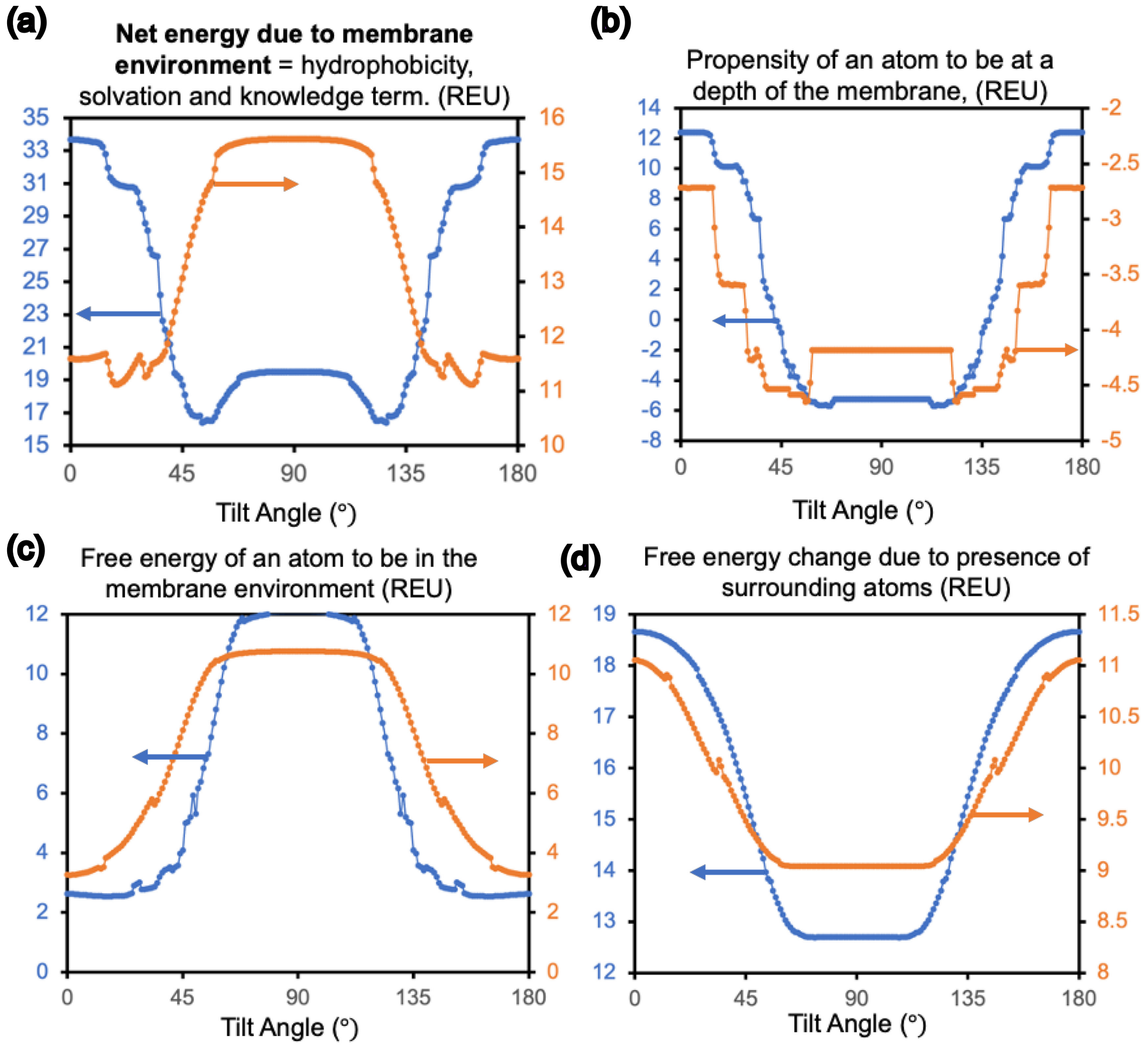

Fig. B. Comparing the energy landscape for WALP25 and WALP35 by M12 at the center of the lipid membrane. The M12 total energy and other membrane-based energy terms were calculated for WALP25 and WALP35 peptides as a function of tilt angle at a depth = 0Å and minimized over rotation angle. The different score terms are as follows: (a) The total energy due to the membrane environment includes hydrophobicity, solvation, and knowledge terms. (b) the knowledge term is a statistical measure of the propensity of a residue to be at a particular membrane depth. (c) hydrophobicity energy and (d) solvation energy.

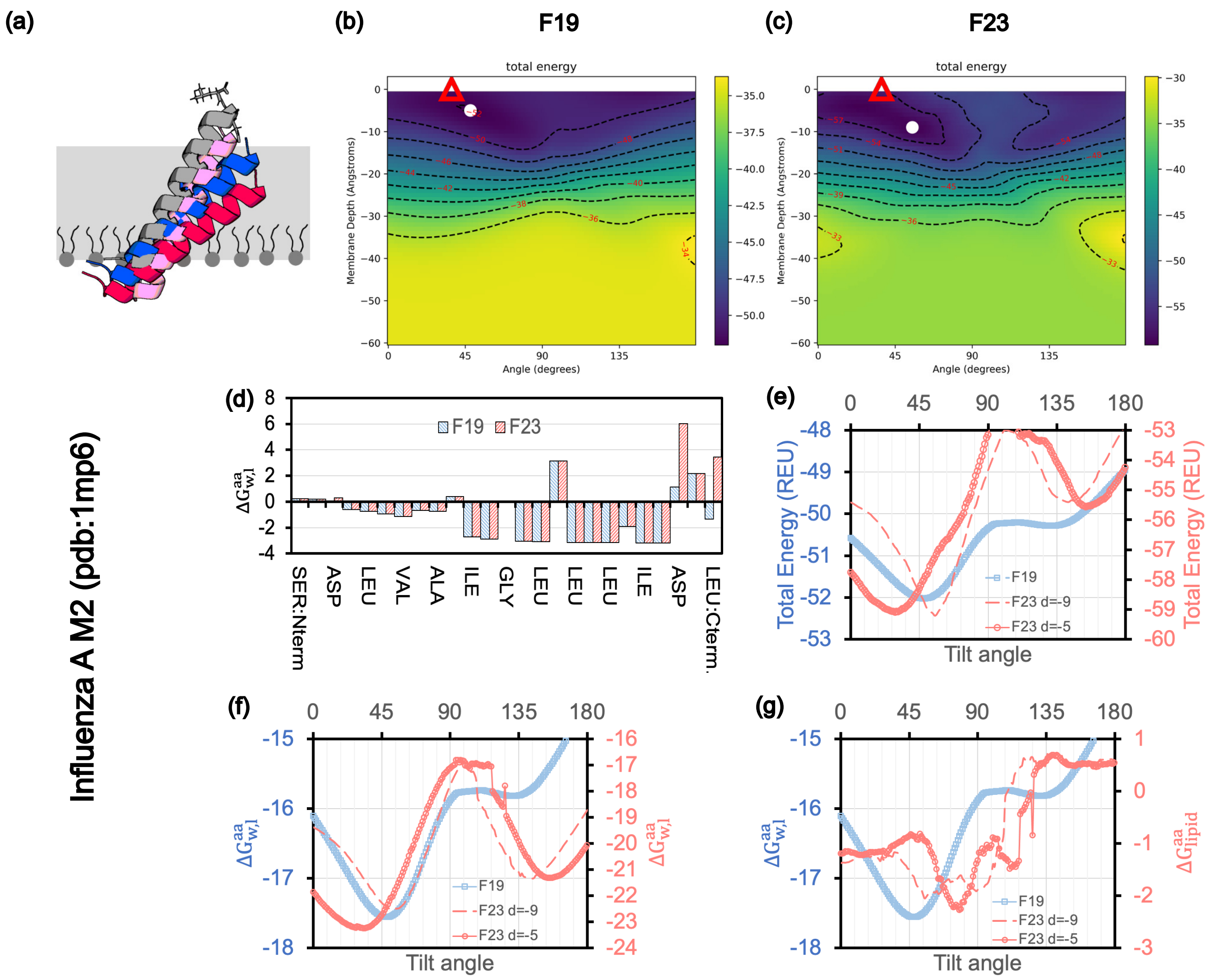

Fig. C. Energy landscape of influenza A M2 peptide (pdb: 1mp6). (a) Comparing the native tilt angle (gray) with that calculated by F19 (blue) and F23(magenta and light pink). The Asp and c-term Leu for which the $\Delta G^{aa}_{w,l}$ is modified by F23 are shown in sticks. The total energy landscape of 1mp6 is shown as calculated by (b) F19 and (c) F23 as a function of depth in the membrane and tilt angle. The energy at each tilt angle is minimized over all azimuthal angles. (d) The per residue $\Delta G^{aa}_{w,l}$ of 1mp6 calculated using F19 and F23 at membrane depth d $= 10$Å, tilt-angle $= 0°$ and azimuthal-angle $= 0°$. The trend of (e) total energy, (f) $\Delta G^{aa}_{w,l}$, and (g) ΔG^{aa}_{lipid} as a function of tilt angles as calculated by F19 at membrane depth d $= 5$Å and F23 at membrane depths d $= 5$ and 9Å.

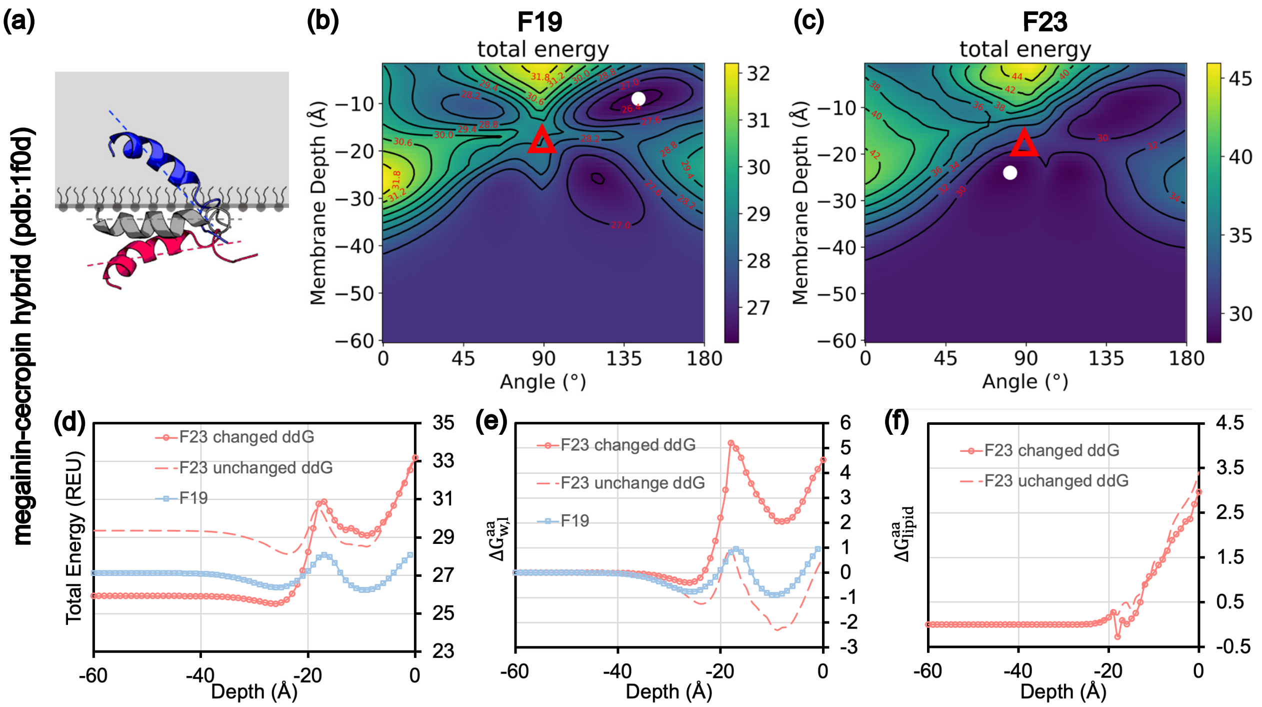

Fig. D. Energy landscape of magainin-cecropin hybrid peptide (pdb: 1f0d). (a) Comparing the native tilt angle (gray) with that calculated by F19 (blue) and F23(magenta). The total energy landscape of 1f0d is shown as calculated by (b) F19 and (c) F23 as a function of depth in the membrane and tilt angle. The energy at each tilt angle is minimized over all azimuthal angles. The trend of (d) total energy, (e) $\Delta G^{\text{aa}}_{w,l}$, and (f) $\Delta G^{\text{aa}}_{\text{lipid}}$ as a function of membrane depth as calculated by F19 and F23 minimized over all tilt-angle and azimuthal-angle.

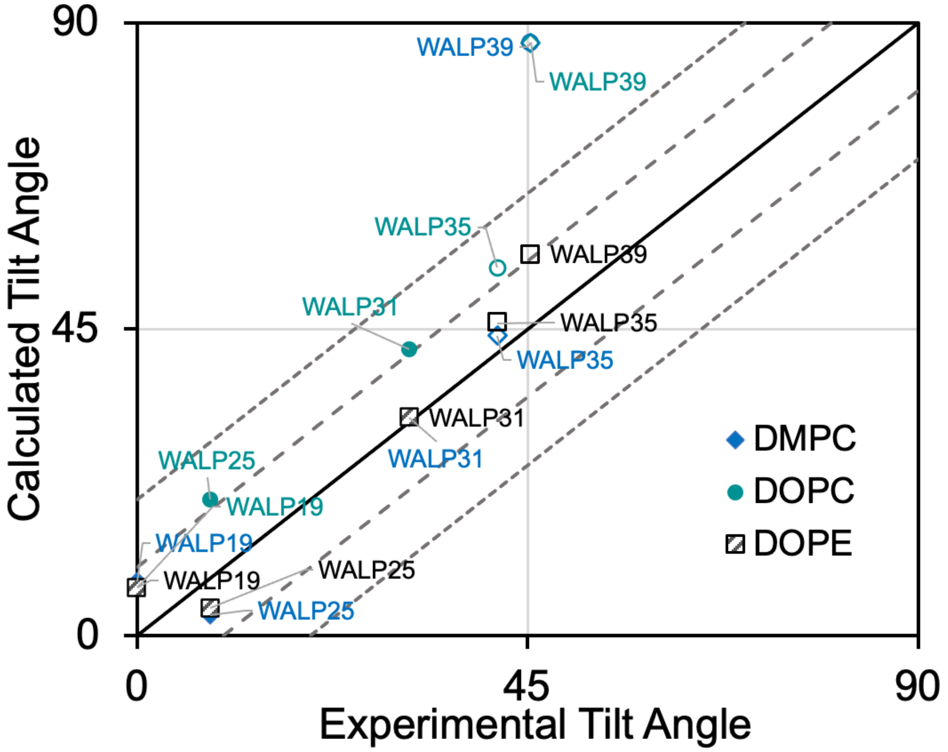

Fig. E. Test1:Orientation of WALP in different lipid layers. Predicted tilt angle of WALP peptides as a function of the number of residues. The WALP peptides are represented as WALPx, where x is the number of residues. The different markers indicate tilt angles calculated using F23 and different lipid types. The experimental tilt angles are measured in the DMPC lipid types. Filled markers present peptides for which experimental results are available, and those for which simulated data is available are shown by unfilled markers.

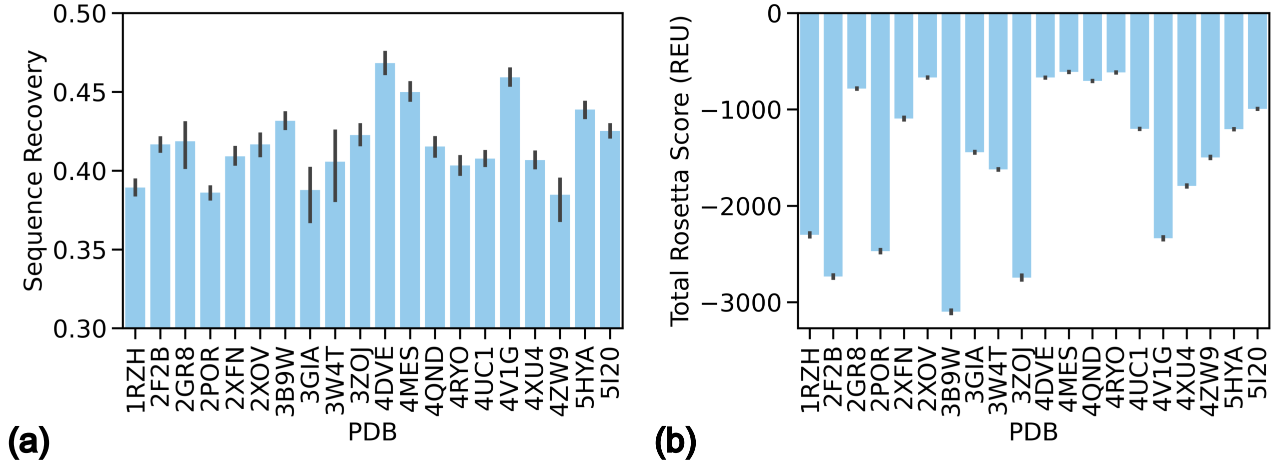

Fig. F. Test6: Variation in the Rosetta designs for a given PDB backbone. (a) Comparing the sequence recovery for a PDB backbone. The bar shows the mean and the error bar shows the variation in the sequence recovery of 50 Rosetta designs for a given PDB. (b) Comparing the total Rosetta score (REU) for a PDB backbone. The bar shows the mean and the error bar shows the variation in the total score of 50 Rosetta designs for a given PDB.

Table A. Electrostatic potential parameters fit from all-atom molecular dynamics data.

Lipid Type	Chain	center C_1	center C_2	center C_3	center C_4	center C_5	Z_c	side A_1	side A_2	side A_3	side A_4	Temp (°C)
DPPG	16:0/16:0	7.83E-05	3.00E-05	-4.94E-03	2.11E-04	9.88E-02	4.02	-0.18	-0.39	20.29	1.58	37
DPPC	16:0/16:0	1.74E-04	2.63E-05	-9.00E-03	1.33E-03	1.47E-01	3.67	-0.18	-0.39	18.46	1.11	37
DPPE	16:0/16:0	5.39E-04	-6.97E-05	-2.41E-02	4.83E-03	3.54E-01	3.49	-0.3	-0.72	25.19	2.25	37
DMPC	14:0/14:0	1.29E-04	-8.36E-06	-7.61E-03	-3.28E-03	1.87E-01	4.12	-0.18	-0.45	15.43	1.03	37
POPC	16:0/18:1	7.15E-05	2.36E-05	-5.30E-03	-1.62E-03	1.49E-01	4.49	-0.19	-0.43	17.19	1.00	37
POPE	16:0/18:1	1.48E-04	-2.15E-05	-8.44E-03	1.12E-03	1.71E-01	3.98	-0.23	-0.5	22.59	1.63	37
POPG	16:0/18:1	4.73E-05	3.28E-05	-4.06E-03	-1.83E-03	7.64E-02	5.21	-0.17	-0.33	18.68	1.24	37
DLPC	12:0/12:0	1.50E-04	-2.47E-05	-7.95E-03	-8.70E-04	2.11E-01	4.03	-0.18	-0.48	13.23	1.22	37
DLPE	12:0/12:0	2.71E-04	1.40E-06	-1.32E-02	2.33E-04	2.69E-01	3.73	-0.24	-0.6	18.51	2.03	37
DLPG	12:0/12:0	1.01E-04	-3.83E-05	-5.66E-03	9.18E-04	1.26E-01	4.05	-0.12	-0.28	13.56	1.03	37
DOPE	18:1/18:1	6.47E-05	8.10E-06	-5.64E-03	-2.50E-05	1.68E-01	4.84	-0.23	-0.5	22.5	1.65	37
DOPC	18:1/18:1	6.01E-05	-7.30E-06	-5.23E-03	2.15E-03	1.42E-01	4.82	-0.18	-0.4	17.23	0.8	37
DOPG	18:1/18:1	3.85E-05	3.23E-05	-3.81E-03	-2.44E-04	5.12E-02	6.06	-0.15	-0.29	18.91	1.11	37

Table B. Equation to calculate the $\Delta G^{\text{atom}}_{w,l}$ transfer energy.

Mutation	Equation	$\Delta\Delta G^{\text{mut}}_{\text{exp}}$(kcal/mol)(1)
A	x_{CH3}	-1.57
C	$x_{\text{CH2}} + x_{\text{SH1}}$	-1.08
D^0	$x_{\text{CH2}} + x_{\text{COO}} + x_{\text{OOC}} + x_{\text{OH}}$	1.38
E^0	$2x_{\text{CH2}} + x_{\text{COO}} + x_{\text{OOC}} + x_{\text{OH}}$	0.07
D^{-1}	$x_{\text{CH2}} + x_{\text{COO}} + 2x_{\text{OOC}}$	6.31
E^{-1}	$2x_{\text{CH2}} + x_{\text{COO}} + 2x_{\text{OOC}}$	6.31
H	$x_{\text{CH0}} + x_{\text{CH2}} + 2x_{\text{aroC}} + x_{\text{Nhis}} + x_{\text{Ntrp}}$	3.19
I	$x_{\text{CH2}} + x_{\text{CH3}}$	-3.12
K	$4x_{\text{CH2}} + x_{\text{Nlys}}$	3.82
L	$2x_{\text{CH3}} + x_{\text{CH2}} + x_{\text{CH1}}$	-3.32
M	$x_{\text{CH3}} + 2x_{\text{CH2}} + x_{\text{S}}$	-2.33
N	$x_{\text{CH2}} + x_{\text{ONH2}} + x_{\text{CNH2}} + x_{\text{NH2O}}$	1.91
P	$x_{\text{Npro}} + 3x_{\text{CH2}}$	-3.09
Q	$2x_{\text{CH2}} + x_{\text{ONH2}} + x_{\text{CNH2}} + x_{\text{NH2O}}$	1.44
R	$3x_{\text{CH2}} + x_{\text{aroC}} + 2x_{\text{Narg}} + x_{\text{NtrR}}$	2.14
S	$x_{\text{CH2}} + x_{\text{OH}}$	0.26
T	$x_{\text{CH3}} + x_{\text{CH2}} + x_{\text{OH}}$	0.21
V	$2x_{\text{CH3}} + x_{\text{CH1}}$	-2.34
W	$3x_{\text{CH0}} + x_{\text{CH2}} + 5x_{\text{aroC}} + x_{\text{Ntrp}}$	-1.95
Y	$2x_{\text{CH0}} + x_{\text{CH2}} + 4x_{\text{aroC}} + x_{\text{OH}}$	-2.66

Table C. Lipid composition parameters for α-helical peptide tilt-angle calculations.

PDB Code	Experimental conditions	Parameters	Reference
1a11	DPC micelles	DLPC	(2, 3)
1mp6	DMPC vesicles	DMPC	(2, 3)
1pje	DOPC:DOPG 9:1	DOPC	(2, 3)
2nr1	DPC micelles	DLPC	(2, 3)
WALP23	DOPC	DOPC	(2, 3)
1f0d	DPC micelles	DLPC	(2, 3)
1f0e	DPC micelles	DLPC	(2, 3)
1f0g	DPC micelles	DLPC	(2, 3)
1hu5	DPC micelles	DLPC	(2, 3)
1hu6	DPC micelles	DLPC	(2, 3)
1hu7	DPC micelles	DLPC	(2, 3)
2mag	DPC micelles	DLPC	(2, 3)
LK-n6	DPC micelles	DLPC	(2, 3)
WALP19	DMPC	DMPC	(4–6)
WALP25	DMPC	DMPC	(5, 6)
WALP31	DMPC	DMPC	(5, 6)
WALP35	DMPC	DMPC	(5, 6)
WALP39	DMPC	DMPC	(5, 6)
AA25	-	DMPC	(6)
AA28	-	DMPC	(6)
AA30	-	DMPC	(6)
AA35	-	DMPC	(6)
AA40	-	DMPC	(6)

References

1. Moon CP, Fleming KG, Side-chain hydrophobicity scale derived from transmembrane protein folding into lipid bilayers. *Proceedings of the National Academy of Sciences* **108**, 10174–10177 (2011).
2. Alford RF, Fleming PJ, Fleming KG, Gray JJ, Protein structure prediction and design in a biologically realistic implicit membrane. *Biophysical Journal* **118**, 2042–2055 (2020).
3. Alford RF, Samanta R, Gray JJ, Diverse scientific benchmarks for implicit membrane energy functions. *Journal of Chemical Theory and Computation* **17**, 5248–5261 (2021) PMID: 34310137.
4. Van der Wel PC, Strandberg E, Killian JA, Koeppe RE, Geometry and intrinsic tilt of a tryptophan-anchored transmembrane -helix determined by 2h nmr. *Biophysical Journal* **83**, 1479–1488 (2002).
5. de Planque MRR, Goormaghtigh E, Greathouse DV, Koeppe RE, Kruijtzer JAW, Liskamp RMJ, de Kruijff B, Killian JA, Sensitivity of single membrane-spanning -helical peptides to hydrophobic mismatch with a lipid bilayer: effects on backbone structure, orientation, and extent of membrane incorporation. *Biochemistry* **40**, 5000–5010 (2001) PMID: 11305916.
6. Sengupta D, Meinhold L, Langosch D, Ullmann GM, Smith JC, Understanding the energetics of helical peptide orientation in membranes. *Proteins: Structure, Function, and Bioinformatics* **58**, 913–922 (2005).

List of Figures

A **WALP35 peptide at the center of the lipid membrane.** (a) The total energy by F23 and $\Delta G^{aa}_{w,l}$ using F19 and F23 calculated as a function of tilt angles. (b) The net energy components due to the presence of the lipid membrane as a function of tilt angle calculated using F19(including $\Delta G_{w,l}$), F23 (including $\Delta G^{\text{aa}}_{w,l}$,$\Delta G^{\text{aa}}_{\text{lipid}}$ and M12 (including hydrophobicity, solvation, and knowledge terms). **WALP39 peptide in the lipid membrane.**(c) The per-residue $\Delta G^{aa}_{w,l}$ calculated using F23 and F19 for WALP39 at a depth = 10Å, tilt-angle=90° and azimuthal-angle = 0°. (d) The total energy by F23 and $\Delta G^{aa}_{w,l}$ using F19 and F23 for WALP39 calculated as a function of tilt angles. (e) The net energy components due to the presence of the lipid membrane at depth = 0Å and as a function of tilt angle calculated using F19, F23 and M12. The arrows show the corresponding axes for the plot. The ones for which arrows are not shown have values represented in the other direction. 2

B **Comparing the energy landscape for WALP25 and WALP35 by M12 at the center of the lipid membrane.** The M12 total energy and other membrane-based energy terms were calculated for WALP25 and WALP35 peptides as a function of tilt angle at a depth = 0Å and minimized over rotation angle. The different score terms are as follows: (a) The total energy due to the membrane environment includes hydrophobicity, solvation, and knowledge terms. (b) the knowledge term is a statistical measure of the propensity of a residue to be at a particular membrane depth. (c) hydrophobicity energy and (d) solvation energy. 3

C **Energy landscape of influenza A M2 peptide (pdb: 1mp6).** (a) Comparing the native tilt angle (gray) with that calculated by F19 (blue) and F23(magenta and light pink). The Asp and c-term Leu for which the $\Delta G^{aa}_{w,l}$ is modified by F23 are shown in sticks. The total energy landscape of 1mp6 is shown as calculated by (b) F19 and (c) F23 as a function of depth in the membrane and tilt angle. The energy at each tilt angle is minimized over all azimuthal angles. (d) The per residue $\Delta G^{aa}_{w,l}$ of 1mp6 calculated using F19 and F23 at membrane depth d = 10Å, tilt-angle = 0° and azimuthal-angle = 0°. The trend of (e) total energy, (f) $\Delta G^{\text{aa}}_{w,l}$, and (g) $\Delta G^{\text{aa}}_{\text{lipid}}$ as a function of tilt angles as calculated by F19 at membrane depth d = 5Å and F23 at membrane depths d = 5 and 9Å. 4

D **Energy landscape of magainin-cecropin hybrid peptide (pdb: 1f0d).** (a) Comparing the native tilt angle (gray) with that calculated by F19 (blue) and F23(magenta). The total energy landscape of 1f0d is shown as calculated by (b) F19 and (c) F23 as a function of depth in the membrane and tilt angle. The energy at each tilt angle is minimized over all azimuthal angles. The trend of (d) total energy, (e) $\Delta G^{\text{aa}}_{w,l}$, and (f) $\Delta G^{\text{aa}}_{\text{lipid}}$ as a function of membrane depth as calculated by F19 and F23 minimized over all tilt-angle and azimuthal-angle. . . 5

E **Test1:Orientation of WALP in different lipid layers.** Predicted tilt angle of WALP peptides as a function of the number of residues. The WALP peptides are represented as WALPx, where x is the number of residues. The different markers indicate tilt angles calculated using F23 and different lipid types. The experimental tilt angles are measured in the DMPC lipid types. Filled markers present peptides for which experimental results are available, and those for which simulated data is available are shown by unfilled markers. . . 6

F **Test6: Variation in the Rosetta designs for a given PDB backbone.** (a) Comparing the sequence recovery for a PDB backbone. The bar shows the mean and the error bar shows the variation in the sequence recovery of 50 Rosetta designs for a given PDB. (b) Comparing the total Rosetta score (REU) for a PDB backbone. The bar shows the mean and the error bar shows the variation in the total score of 50 Rosetta designs for a given PDB. 7

List of Tables

A Electrostatic potential parameters fit from all-atom molecular dynamics data. 8
B Equation to calculate the $\Delta G_{w,l}^{\text{atom}}$ transfer energy. 8
C Lipid composition parameters for α-helical peptide tilt-angle calculations. 9
