# Supplementary material for: Implicit model to capture electrostatic features of membrane environment
Source: PLoS Comput Biol. 2024 Jan 22;20(1):e1011296. doi: 10.1371/journal.pcbi.1011296 (PMC10833867; doi:10.1371/journal.pcbi.1011296)
Supplement: Attachment — Submitted filename: Rev_PCOMPBIOL-D-23-00999.pdf [file pcbi.1011296.s002.pdf]

In this article, the authors have developed a new implicit energy function, Franklin2023 (F23), as an extension of the Franklin2019 (F19) energy function. In the previous article [ref. 37], the authors introduced a 12-test benchmark for the energy function, which was tested on F2019 and identified areas for potential improvement. In this article, several of these suggestions have been implemented, particularly focused on improvements in the representation of membrane electrostatics and the correction of water-to-lipid transfer energies for Glu and Asp residues. The F23 function was validated through several tests, and the results were compared with F2019 and other energy functions from the literature.

It is evident that a significant disparity exists between our current understanding of the structures (and function) of soluble and membrane-related proteins. This underscores the imperative to advance methods for studying proteins within lipid-rich environments. A promising approach lies in implicit energy functions that enable fast calculations of free energies, potentials and experimentally measurable quantities such as tilt angles, and are also very valuable tools in sequence design. Therefore, the topic and methodology presented in this article are very relevant and of significant interest both to the readers of PLOS Computational Biology and to the wider scientific community.

The article is well-written, with effective presentation of methods and results. It is, in my opinion, ready for publication, with only minor corrections, which are listed below, along with a few comments:

1. Please enhance the quality of some figures and add missing details to the captions for the benefit of readers. a) In Figures 4 and 5, some peptide names are partially missing or overlapped. b) It is unclear what the lines in Figure 4 represent. Please add descriptions to the caption. c) Figure 6 is referred to as Figure 6b in the text, and it would be useful to have a more detailed explanation of Figure 6 data in the caption. d) In Figure 9, specify what the empty and filled symbols represent. e) It would be useful to provide a clearer explanation of what the data in Figure 10 represents. f) Please add a more detailed description of Figure S1 data.

2. Please check if the numbering of tests in the final table is accurate.

3. The statement about the lower hydrophobicity of polar residues contributing to a more accurate prediction of the tilt angle and its confirmation in Fig. S1e is a bit puzzling to me. Could the authors rewrite this sentence to make it clearer.

4. Regarding the last test aimed at the protein design, it is not optimal that F23 performed worse than F19 (or nearly the same). However, the improvement in the percentage of sequence recovery for surface-exposed residues, in contrast to buried residues, is promising. Could this indicate a potential path for improvement, possibly by adjusting the representation of the dielectric constant of the bilayer?

5. It is interesting to note that the improvements in predicting stability and design performance were not observed with F2023 as probably expected. The addition of terms to enhance the electrostatic features of the membrane should ideally lead to improvements in all tests. This result may suggest that some essential aspects are still missing or that the included terms fail to represent certain critical contributions. Please could you comment on this in the article.

6. The authors have outlined future directions for energy function development, which involve

adding terms to provide an even more detailed description of the features of the lipid environment, such as pH effects. Does this imply that the authors consider F23 as a good foundation upon which to build by adding new terms?
