# Supplementary material for: Implicit model to capture electrostatic features of membrane environment
Source: PLoS Comput Biol. 2024 Jan 22;20(1):e1011296. doi: 10.1371/journal.pcbi.1011296 (PMC10833867; doi:10.1371/journal.pcbi.1011296)
Supplement: Attachment — Submitted filename: Response_to_Reviewer_comments_final.pdf [file pcbi.1011296.s003.pdf]

Reviewer's Responses to Questions

Editor comment:

Thank you very much for submitting your manuscript "Implicit model to capture electrostatic features of membrane environment." for consideration at PLOS Computational Biology. As with all papers reviewed by the journal, your manuscript was reviewed by members of the editorial board and by several independent reviewers. The reviewers appreciated the attention to an important topic. Based on the reviews, we are likely to accept this manuscript for publication, providing that you modify the manuscript according to the review recommendations.

Here is an additional comment for your consideration based on my own reading (Nir Ben-Tal):

The literature survey includes recent publications. But continuous solvent models as well as coarse-grained models of peptide-membrane interactions date way back, into the previous millennium. The manuscript completely neglects to refer to these. For example, Turkan Haliloglu and myself have published a lot on this. And Skolnick, Efremov, Biggin, Sansom,... I'm giving here two references of my own, just because they are easy for me to find, but they refer to many relevant works also by others. Plenty to read... The authors may want to refer to some of the old works that outlines ideas that are now implemented more accurately. But please feel free to completely ignore this comment. Maybe I'm biased.

1) https://www.sciencedirect.com/science/article/abs/pii/S106358230252010X

2) https://www.ncbi.nlm.nih.gov/pmc/articles/PMC3394254/

End of comment.

Response to editor

We appreciate the editor for introducing us to the literature describing the contribution to continuum solvent and coarse-grained models of membrane-associated peptides, Based on the papers, we have made the following changes:

Change in the manuscript (Page 2, line 46)

Citations for continuum models. (Ben-Tal, Ben-Shaul, et al., 1996; Ben-Tal, Honig, et al., 1996; Kessel et al., 2000; Schlenkrich et al., 1996)

Change in the manuscript (Page 19, line 505)

Additional citations for lipid demixing (Haleva et al., 2004), (Heimburg et al., 1999)

Response to reviewers

We thank the editor and reviewers for thoughtful comments on our work. We have revised the manuscript text, figures, and supplementary information to accommodate all the suggestions. The changes in the manuscripts are highlighted in blue. The following are the detailed responses addressing each comment from the editor as well as the reviewers.

Reviewer #1:

In this work, the authors developed an implicit membrane model (F23) by implementing modifications to a prior model (F19). Modifications include a mean field electrostatic potential based on the atomistic simulations, position dependent dielectric constant term along the membrane normal and a modified energy term for the water to bilayer partitioning for Asp and Glu. Overall, they obtained a moderate improvement over the F19 model, especially for the orientation of membrane peptides/proteins. The most significant addition of the model is to develop the mean field potential using different lipid types, which can allow to study proteins in lipid models with various head groups, saturation level, hydrocarbon chain length. This paper is a valuable contribution to membrane model development and will be of interest for PLOS Computational readers. Below, I listed my comments for improving the manuscript, mostly about providing more computational details to ensure reproducibility.

1- Figure 1 shows the electrostatic potentials based on the atomistic simulations. However, no details were provided about these simulations. I understand that they came from a prior work, an explanation for confirming this would be good. But, otherwise, if the atomistic simulations are part of this work, simulation details should be provided (system size/preparation, simulation time, ensemble, force field, integrator etc.).

We have expanded the section on derivation of electrostatic energy terms based on lipid type by adding simulation details.

Change in Manuscript (line 122, Page 4):

"Following our previous work, MD trajectories used in this analysis were generated from all-atom molecular dynamics simulations of phospholipids, water, and salt. The simulations were performed using the NAMD molecular dynamics engine at a constant pressure of 1 atm and a temperature of 37°C. The CHARMM36 force field was used for lipid and TIP3 model for water. See Alford et al., 2020 methods section for further details."

2- For the tilt angle analysis (Figure 4), it is not clear how the lowest energy orientations of peptides were obtained (lines 204-205). Were they obtained by a Monte Carlo sampling, or were any MD simulations performed? Was a previously established protocol used, or they have developed a new protocol? More details about how these orientations were predicted would be good for reproducibility of the data.

We thank the reviewers for pointing out the missing details of tilt angle prediction. We have now added the following lines.

Change in Manuscript (line 226, Page 8):
"Following our prior work (Alford et al., 2021), we used a protocol that samples all possible orientations of the peptide relative to the implicit membrane within ± 60Å of the bilayer center (d), tilt angles relative to the membrane normal (θ) between $\pm 180°$, rotation angles relative to the principal helical axis (ϕ) between $0 - 360°$. The global energy minimum of all sampled positions is defined as the most stable predicted orientation."

3- Also, which initial structures were used for both AAx and WALP peptides?
The structures for AA_x and WALP peptides of different lengths are both modeled as ideal $\alpha -$helices with $\psi = -47°$ and $\phi = -57°$. We are adding the following line for WALP and AA_x peptides in the manuscript.
Change in the manuscript (Page 9, line 248):
"The WALP for this test are modeled as ideal $\alpha -$helices with $\psi = -47°$ and $\phi = -57°$."

4- For predicting orientation of the peptides (AAx and WALP, TM and adsorbed), which electrostatic potential was used for F23, from which lipid model, what length, and head group? Authors mentioned using DLPC for WALP peptides (lines 221-223), but it is not clear which lipid type was used for other peptides/proteins. Peptide orientations will vary significantly according to the membrane width, so the membranes (lipid type) used in the experiments/simulations and in their models should be provided (maybe in a table) for making a meaningful comparison.
In our previous submission we had used DOPC lipids for calculating the tilt angles for WALP peptides. However, we realized that DMPC lipids were used for the experimental results, so we have now recalculated the results in DMPC. We have revised Figure 4b.

The experimental solid state NMR measurements by Van Der Wel et al., 2002 for WALP19 were collected in three different bilayer-forming lipids, DLPC, DMPC and DOPC. Interestingly, in these experiments the tilt angle was insensitive to the lipid type. Infrared spectroscopic measures of tilt angle for WALP19-31 are presented in De Planque et al., 2001 for DMPC (14:0-PC) lipid layer. Sengupta et al. reported the tilt angle for WALP19 as ~0, whereas Planque et al. reports it to be close to 10 degrees. We have updated the manuscript to clearly mention the lipid model as follows.
Change in the manuscript (Page 9, line 250):
"In this test, we calculate the tilt angle of WALP peptides of different lengths in DMPC lipid layers."
Change in the manuscript (page 9, line 239):
"Due to the lack of any lipid type mentioned in the 5-slab model, in this test, we calculated the tilt angles using DLPC for both F19 and F23."
Figure 4b is updated in the manuscript.

5- The authors mentioned that the model is fast-to-compute. It would be good to add some benchmarking about the computing time performances.

Ulmschneider & Ulmschneider, 2009 undertook a comprehensive analysis of sampling efficiency in explicit and implicit membrane environments while studying peptide folding simulations. They compared the complete folding process of WALP16 peptide using an implicit membrane model based on generalized Born method, an explicit di-palmitoyl-phosphatidyl-choline lipid bilayer and an octane slab membrane mimic. A quantitative assessment of sampling efficiency, utilizing a wide range of performance metrics revealed that the implicit membrane models were at least 2 orders of magnitude more efficient than the simplest explicit model, namely, the octane membrane mimic. The superior efficiency of the implicit models can be attributed to two key factors. First, the elimination of solvent and lipid degrees of freedom reduces the number of atoms to be treated explicitly by ~80%. This significantly reduces the cost per time-step when compared to explicit solvent or lipid representation. Additionally, the mean-field nature of the solvent allows for substantially accelerated dynamics per time-step so that relevant thermodynamic states, leading to sampling of relevant thermodynamic states of the biomolecules compared to solvents or lipids. Of course, we recognize that both solvent and lipid play a crucial role, and developing an implicit model that accurately represents the membrane environment is a challenging endeavor.
Change in the manuscript (Page 2, line 47):

To compare the computing time performances for an implicit and explicit membrane model, Ulmschneider and Ulmschneider (Ulmschneider & Ulmschneider, 2009) undertook a comprehensive analysis of sampling efficiency of WALP16 peptide folding using a GB based implicit membrane model, an explicit DPPC lipid bilayer and an octane slab membrane mimic. A quantitative assessment of sampling efficiency, utilizing a wide range of performance metrics revealed that the implicit membrane models were at least two orders of magnitude more efficient than the octane membrane mimic, simplest of the two explicit models.

Reviewer #2:

Summary:
The authors present updates to the Rosetta membrane score function that include two new score terms, an electrostatic energy term and a membrane dependent dielectric constant, and a modification to an existing membrane dependent score term. They show results from multiple tests for the previous score functions and the new score function. The paper is well written and organized. The improvements to the Rosetta membrane score function adds an important contribution to computational membrane protein structure prediction. Some of the choices the authors made could be justified or explained in more detail. It is unclear from the data presented how variable the results for different tests are when comparing independent runs. The work could benefit from a more detailed supplementary method section or protocol captures.

Major Comments:

• The authors should provide more details on how weights were optimized for the three membrane dependent energy terms. The authors say the weights were optimized to maximize the correlation coefficient for experimentally measured ddGs and then cite two papers. One paper is the hydrophobicity scale from Moon and Fleming and the

other is a review on the Poisson-Boltzmann equation. Is that the correct reference, reference 35 on line 171 in the “Integration of new terms into F23” section? What protocol was used to calculate the predicted ddGs with F23 that were used for the weight optimization? Are there any concerns with fitting the weights using the same experimental data that the transfer energy from water to bilayer term is based on? How/why were Gly, Ala, Pro, Asp, and Glu chosen to be excluded for overfitting? Is there any way to optimize these weights using more than one task?
We thank the reviewer for pointing this, We have added more details on the protocol to find the weights. Additionally, we have added the reasons for removing the residues.

Change in the manuscript (line 183, page 7):
We solved the simultaneous equations for the three unknown positive weights to maximize the correlation coefficient for the experimentally measured $\Delta\Delta G_{mut}$ for amino acids at the center of OmpLA protein (Moon & Fleming, 2011) and mutations to aromatic side chains (Phe, Trp, Tyr) (McDonald & Fleming, 2016). The resulting weights were $w_{w,l}$ = 1, w_{lipid} = 0.128 and w_{dielec} = 0.01, achieved using L-BFGS-B algorithm within bounds of [0,1]. Our choice of these bounds were to avoid negative weights and extremely large positive weights, while recognizing potential biases due to the bounds and more data points at the center of the membrane. We omitted the matrix rows for Ala due to its role as a host residue for all experimental mutations, Pro to avoid steric clashes resulting in large values, Asp and Glu residues because the experimental values were measured at low pH.

• How much variation is there in ddG predictions? Could multiple runs be done with mean and standard deviation be shown in scatter plots?
We have repeated the ddG predictions for each protein (OmpLA and PagP) 10 times. However, they are so small that they are covered by the markers. So we report the standard deviation in text.
Change in the manuscript (page 13, line 358)
To assess the uncertainty of the predicted ΔΔG, we conducted 10 repeated calculations, resulting in standard deviations of 0.008 (F23, OmpLA), 0.016 (F23, PagP), 0.086(F19, OmpLA) and 0.009 (F19, PagP).

• How consistent are design results? Were these ran multiple times or just once?
We generated 50 design sequences for the fixed backbone using Rosetta’s Monte Carlo fixed-backbone design protocol. We then chose the lowest scoring sequence for further analysis. For clarification, we have added the following line in the manuscript in line 413.

Change in the manuscript (page 14, line 386):
“For each backbone, we generate 50 designed sequences for a given backbone conformation and chose the lowest scoring sequence for further analysis.’’

Change in the manuscript (page 14, line 403):
“To estimate the variation of designed sequences for a given backone, Figure S6a-b show the mean and standard deviation of sequence recovery and total score of all 50 designs for top 20 performing PDB backbones.’’

Minor Comments:
• F19 has 13 different lipid options. It is unclear if F23 has the same lipid options or only some of them.
F23 has the same lipid options as F19. For clarification, we have added a table in SI and the following line in the manuscript.
<u>Change in the manuscript (page 4, line 106):</u>
"We have calculated the electrostatic potential for all the 13 lipid layer options available with F19 and are listed in Table S1.''

Table S1 is added to SI.
• Figure 3B: Were the large shifts in dGw,l for COO and OOC in F23 compared to F19 expected? Is there any significance that OOC for F23 is now almost the same as in IMM1?
In our previous work, (Alford et al., 2020, 2021) we have used the $\Delta G_{w,l}^{aa}$ for protonated states of Asp and Glu (D^0 and E^0) from MF scale. In our current work, we have used the corrected equations for the protonated states (D^0 and E^0), in addition to using extrapolated values for negatively charged states of Asp and Glu (D^{-1}, E^{-1}) from WW scale details of which is described in Page 6, lines 166. This change in $\Delta G_{w,l}^{D^0} = 1.38 \rightarrow \Delta G_{w,l}^{D^{-1}} = 6.31$ and $\Delta G_{w,l}^{E^0} = 0.07 \rightarrow \Delta G_{w,l}^{E^{-1}} = 6.31$ has caused the large shift in COO and OOC.

We can summarize the changes as follows:
(Alford et al., 2020, 2021)
$x_{CH2} + x_{COO} + 2x_{OOC} = 1.38$
$2x_{CH2} + x_{COO} + 2x_{OOC} = 0.07$

Current publication:
$x_{CH2} + x_{COO} + x_{OOC} + x_{OH} = 1.38$
$2x_{CH2} + x_{COO} + x_{OOC} + x_{OH} = 0.07$
$x_{CH2} + x_{COO} + 2x_{OOC} = 6.31$
$2x_{CH2} + x_{COO} + 2x_{OOC} = 6.31$

<u>Change in the manuscript (Page 7, line 173):</u>
Table S3 shows all the equations and values of $\Delta G_{w,l}^{aa}$.
- In the introduction the authors mention a previously developed 12-test benchmark. Could the authors either show results, even just as part of the supplementary materials, for all the 12 test or provide rational behind the test they chose to show? For example, there no docking results shown.

We appreciate the reviewer's suggestion of showing the results for all 12 benchmark tests in the supplementary. However, the paper is already long, and we did not want to lengthen it further, so we focused on a fewer test cases. We have decided to follow this paper up with detailed analysis of performance of Franklin2023 on the other tests. For docking, we are finishing up a dedicated manuscript which can be expected by the end of 2023, where we are investigating a flexible backbone membrane protein docking and

compare its results on bigger membrane protein complex dataset for both Franklin2019 and Franklin2023.

• What protocol is used to determine the tilt angle for peptides?

We thank the reviewers for pointing out the missing details of tilt angle prediction. For improved understanding, we have now added the following lines in the manuscript.

Change in Manuscript (line 226, Page 8):

"Following our prior work (Alford et al., 2021), we used a protocol that samples all possible orientations of the peptide relative to the implicit membrane with ± 60Å of the bilayer center (d), tilt angles relative to the membrane normal (θ) between $\pm 180°$, rotation angles relative to the principal helical axis (ϕ) between $0 - 360°$. The global energy minimum of all sampled positions is defined as the most stable predicted orientation."

• I find it odd that in the polyalanine tilt angle test M12 does not recapitulate the results from the 5-slab model, but the argument for why M12 matches the 5-slab model for the WALP peptides is they are based on the same hydrophobicity scale.

Although the hydrophobicity of both M12 and the 5-slab model are based on the Wimley White hydrophobicity scale, M12 has an additional heuristic term based on the probability of finding a residue at a given depth of the membrane. For WALP peptides, it penalizes placing end TRP residues in the membrane, whereas for ALA residues in the polyalanines, the term forces them to be in the membrane.

Change in the manuscript (line 276, page 9):

However, hydrophobicity alone cannot be the reason, as otherwise, the tilt angles for polyalanines would have been similar. M12 uses a knowledge term based on the probability of finding a residue at a given depth of the membrane, which penalizes the Trp residues in the membrane but not the Ala residues.

• Were different lipid compositions used for Test 2?

Yes, we have used different lipid layer compositions for tilt angle calculation because the experimental results are from NMR experimental papers using different lipid compositions. For example, we have used DMPC for 1mp6, DOPC for 1pje and WALP23. Peptides for which the experimentally used lipid layer is outside the scope of this paper, DLPC. For example, 1a11 and 2nr1 used DPC micelles, we have used our default lipid layer. The experimental reference tilt angle of all adsorbed peptide (viz. 1hu5, 1hu6, 1f0d, 1f0e and 1f0g) targets were measured by solution NMR in dodecylphosphocholine micelles. We have used DLPC to calculate the tilt angle like transmembrane peptides. For the reader's better understanding, we have added a supplementary table stating the experimental condition and parameters used.

Change in the manuscript (Line 289, page 10) :

"The different lipid types used for experiments and the parameters used for this test are listed in Table S2."

Table S2 is added in the SI.

- How much does the lipid composition effect the results of tilt angle predictions?

The lipid composition affects the tilt angles significantly due to the interaction of electrostatic field due to the lipid head group and the residues (ΔG_{lipid}) and the hydrophobic interaction with the lipid bilayer ($\Delta G_{w,l}$). We have now added the following Figure *1* in the SI.

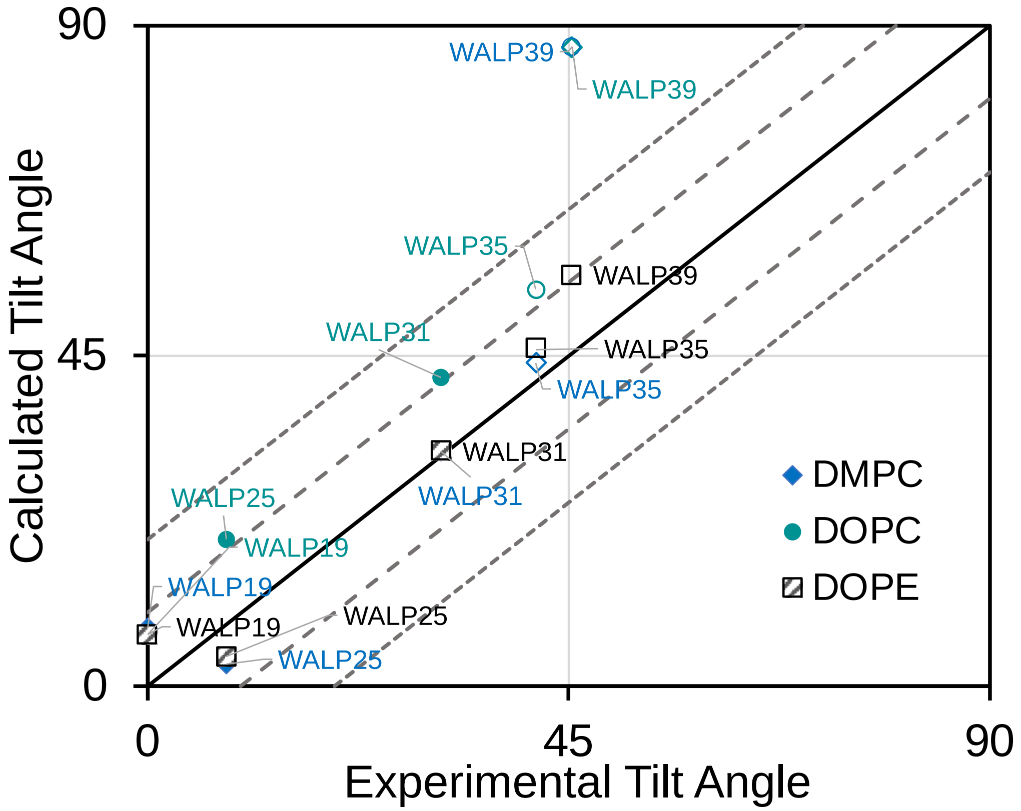

Figure 1 **Test1: Orientation of WALP peptides in different lipid layers.** Predicted tilt angle of WALP peptides as a function of the number of residues. The WALP peptides are represented as WALPx, where x is the number of residues. The different markers indicate tilt angles calculated using F23 and different lipid types. The experimental tilt angles are measured in the DMPC lipid types. Filled markers present peptides for which experimental results are available, and those for which simulated data is available are shown by unfilled markers.

Change in the manuscript (Page 9, Line 264):
"To demonstrate the impact of lipid composition, Figure S5 displays calculated WALP tilt angles using F23 in three different lipid compositions.''

- Fix quality of figure S2 so the faint outline around the plots are not seen

We have removed the faint outlines in the revised manuscript.
Change in the manuscript:
Revised Figure S2.

- What is the little blue dot in Figure 7A above the F23 datapoint for Lysine?

We appreciate the reviewer for raising this point. It was a dot which was added in our editing software. In the revised manuscript we have removed the dot.

Change in the manuscript:

Revised Figure 7.

• In Figure 7, could the authors include which color line and text correspond to which energy function.

We have rewritten the description in the figure label.

Change in the manuscript (Revised Figure 7 label):

The red and blue dashed lines represent the linear best fit lines for values predicted by F23 and F19 respectively. The equations for the linear best fit lines for F23 and F19 are shown in the plots in red and blue respectively.

• In Figure 9, datapoints are shown for energy functions not previously introduced.

We have now added a line to describe the previous energy functions.

Change in the manuscript (line 405, page 14):

The soluble protein energy function (R15) and other previous implicit membrane energy function which was parameterized from the behavior of side-chain analogs in organic solvents (M07) lagged in sequence recovery.

Reviewer #3:

Summary

In this article, the authors have developed a new implicit energy function, Franklin2023 (F23), as an extension of the Franklin2019 (F19) energy function. In the previous article [ref. 37], the authors introduced a 12-test benchmark for the energy function, which was tested on F2019 and identified areas for potential improvement. In this article, several of these suggestions have been implemented, particularly focused on improvements in the representation of membrane electrostatics and the correction of water-to-lipid transfer energies for Glu and Asp residues. The F23 function was validated through several tests, and the results were compared with F2019 and other energy functions from the literature.

It is evident that a significant disparity exists between our current understanding of the structures (and function) of soluble and membrane-related proteins. This underscores the imperative to advance methods for studying proteins within lipid-rich environments. A promising approach lies in implicit energy functions that enable fast calculations of free energies, potentials and experimentally measurable quantities such as tilt angles, and are also very valuable tools in sequence design. Therefore, the topic and methodology presented in this article are very relevant and of significant interest both to the readers of PLOS Computational Biology and to the wider scientific community.

The article is well-written, with effective presentation of methods and results. It is, in my opinion, ready for publication, with only minor corrections, which are listed below, along with a few comments:

1. Please enhance the quality of some figures and add missing details to the captions for the benefit of readers. a) In Figures 4 and 5, some peptide names are partially missing or overlapped. b) It is unclear what the lines in Figure 4 represent. Please add descriptions to the caption. c) Figure 6 is referred to as Figure 6b in the text, and it would be useful to have a more detailed explanation of Figure 6 data in the caption. d) In Figure 9, specify what the empty and filled symbols represent. e) It would be useful to provide a clearer explanation of what the data in Figure 10 represents. f) Please add a more detailed description of Figure S1 data.

Change in the manuscript:

**Figure #**	**Changes made in the manuscript**
Figure 4a	Overlapping labels for markers are now removed and relabeled.
Figure 4b	Tilt angles were rerun for DMPC instead of DOPC. Overlapping labels for markers are now removed and relabeled.
Figure 5(a-b)	Overlapping labels for markers are now removed and relabeled.
Figure 6	Each row is now labeled as a,b,c.
Figure 7	The blue dot in (a) is removed. Description of the dashed lines are added in the caption of Figure 7.
Figure 9	Changes in caption mentioning differences in open and filled circles.
Figure 10	Different residue types are now added in the pie-chart.

2. Please check if the numbering of tests in the final table is accurate.
We thank the reviewer for pointing this out. We have now corrected the test number.
Change in the manuscript:
Table 1 is now updated.

3. The statement about the lower hydrophobicity of polar residues contributing to a more accurate prediction of the tilt angle and its confirmation in Fig. S1e is a bit puzzling to me. Could the authors rewrite this sentence to make it clearer.

We thank the reviewer for pointing this out. We mistakenly cited S1c as S1e. We have now rewritten the explanation in line 236, page 8.

4. Regarding the last test aimed at the protein design, it is not optimal that F23 performed worse than F19 (or nearly the same). However, the improvement in the percentage of sequence recovery for surface-exposed residues, in contrast to buried residues, is promising. Could this indicate a potential path for improvement, possibly by adjusting the representation of the dielectric constant of the bilayer?
We thank the reviewer for pointing this out. We have optimized the weight of the energy terms using only $\Delta\Delta G_{mut}$, which is biased to more observations at the center of the membrane and for β barrel proteins (OmpLA) which resulted in quite a low weight for the dielectric term. We can use rigorous optimization techniques to characterize the fitting parameters and weights to reduce double counting of factors and minimizing errors of more generalized experimental measurements such as design.
<u>Change in the manuscript (Page15, line412):</u>
This improvement in the sequence recovery of surface exposed residues in contrast to buried by F23 may indicate a potential path for improvement particularly weights of the dielectric constant term which is lower relative to others.

5. It is interesting to note that the improvements in predicting stability and design performance were not observed with F2023 as probably expected. The addition of terms to enhance the electrostatic features of the membrane should ideally lead to improvements in all tests. This result may suggest that some essential aspects are still missing or that the included terms fail to represent certain critical contributions. Please could you comment on this in the article.
As the reviewer pointed out, the results for the benchmark tests show that, relative to F19, F23 has improved the ability of implicit membrane models to capture more relevant features of membranes. We think some of the potential directions for improvement are as follows:

1. Adding the effect of pH to account for membrane induced effect of pKa.
2. Using more robust techniques to determine the weights for the terms and optimize the fitting parameters.
3. Adding mixed and asymmetric lipid layers
4. Adding deformation of the lipid interface as it is perturbed by charged amino acids.

We have added the following lines to enhance understanding in the manuscript.
<u>Change in the manuscript (Page 19, line 498)</u>:
However, F23 did not improve predictions of stability and design performance as expected, possibly due to an incomplete representation of the membrane environment in the model.

6. The authors have outlined future directions for energy function development, which involve adding terms to provide an even more detailed description of the features of the lipid environment, such as pH effects. Does this imply that the authors consider F23 as a good foundation upon which to build by adding new terms?

Yes, we think F23 as a good foundation model as it includes more detailed electrostatic terms which are important in characterizing the pH environment. In our next project when we add these features, we will add an extensive benchmark dataset to test our protocols.

Change in the manuscript (Page 19, line 506):
F23 can serve as a good foundation model for including pH or lipid mixtures, as it includes additional electrostatic features and more characteristics of the lipid features.
Change in the manuscript (Page 19, line 510):
Approaches such as (Woods et al., 2023) those that include a defined curvature or elastic bending constants of lipid membranes (Marsh, 2006) along with other robust models can serve as foundation models for this purpose.
